# Possible deep connection between volcanic systems evidenced by sequential assimilation of geodetic data

**DOI:** 10.1038/s41598-018-29811-x

**Published:** 2018-08-03

**Authors:** Mary Grace Bato, Virginie Pinel, Yajing Yan, François Jouanne, Jean Vandemeulebrouck

**Affiliations:** 1 Université Grenoble Alpes, Université Savoie Mont Blanc, CNRS, IRD, IFSTTAR, ISTerre, 38000 Grenoble, France; 2 Université Savoie Mont Blanc, LISTIC, 74000 Annecy, France

## Abstract

The existence of possible deep connections between nearby volcanoes has so far only been formulated on the basis of correlation in their eruptive activities or geochemical arguments. The use of geodetic data to monitor the deep dynamics of magmatic systems and the possible interference between them has remained limited due to the lack of techniques to follow transient processes. Here, for the first time, we use sequential data assimilation technique (Ensemble Kalman Filter) on ground displacement data to evaluate a possible interplay between the activities of Grímsvötn and Bárðarbunga volcanoes in Iceland. Using a two-reservoir dynamical model for the Grímsvötn plumbing system and assuming a fixed geometry and constant magma properties, we retrieve the temporal evolution of the basal magma inflow beneath Grímsvötn that drops by up to 85% during the 10 months preceding the initiation of the Bárðarbunga rifting event. We interpret the loss of at least 0.016 km^3^ in the magma supply of Grímsvötn as a consequence of magma accumulation beneath Bárðarbunga and subsequent feeding of the Holuhraun eruption 41 km away. We demonstrate that, in addition to its interest for predicting volcanic eruptions, sequential assimilation of geodetic data has a unique potential to give insights into volcanic system roots.

## Introduction

The rate of magma supply to volcanic systems which fundamentally controls the eruptive activity is a determinant piece of information mostly retrieved by geodesy and/or gas measurements. However, this key input remains difficult to constrain. One reason is that the ability of geodetic observations to detect or quantify magma accumulation decreases with the increasing depth of storage zones involved. Despite this flaw, geodesy sometimes in combination with gas measurements has been essential to estimate magma flux entering subsurface reservoirs, proving that this supply was most probably occurring by pulse or surge of magmas^1,2^. Indeed, this behavior is consistent with the observation that long-term pluton growth rates, inferred from isotopic studies, are much smaller than the minimum rates of magma supply required to ensure magma transfer through dykes^3^. Despite this known transient behavior in deep magma supply, classical methods used so far to invert geodetic data always consider steady-state systems with constant basal inflow^4^.

Another open question related to magmatic sources concerns their spatial extent at depth, whether or not a common deep source can be shared by different volcanic systems or distinct magmatic sources can be mechanically connected at depth. Geochemical arguments based on major element compositions or ratios as well as isotopic compositions are commonly used to discriminate samples from different volcanic systems and address the question of the lateral extension of volcanic roots^5,6^. More recently, thanks to the improvement of deformation data spatial coverage brought by satellite radar interferometry, geodesy has provided some significant insights into the lateral extent of magmatic domains^7,8^. Rifting areas where long dikes are emplaced, represent ideal context to track lateral connections between nearby volcanic systems.

Here, we propose a new methodology suitable to retrieve magma supply changes from temporal series of geodetic data even with limited amount of spatial information, allowing us to evidence a possible connection between two nearby volcanic systems in the Icelandic Eastern Volcanic Rift Zone. While several studies have used real geodetic data and applied variants of Kalman Filter as an optimization or statistical interpolation tool to solve problems in volcanology in the past^9–11^, this study is the first one to apply sequential data assimilation based on a dynamical model as proposed by ref.^12^ using a real dataset recorded on a volcano.

## Bardarbunga and Grimsvotn volcanoes: Related deformation before the 2014–2015 eruptive activity

**Figure 1:**
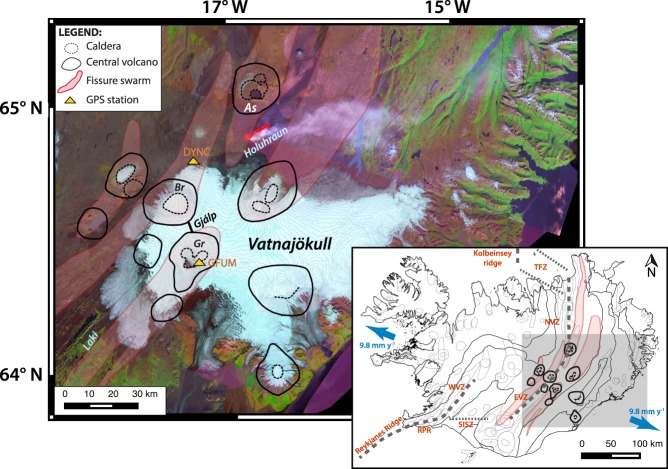
Landsat-8 image taken on 06 September 2014, showing the principal volcanoes and fissure swarms (e.g. Bárðarbunga (Br), Grímsvötn (Gr) and Askja(As)) near the Vatnajökull icecap. The image is based on the mosaicked data from the National Land Survey of Iceland^45,46^). Fissure eruptions of Laki (1783–1784) and Gjálp (1996) as well as the on-going Holuhraun eruption when the image is captured are also presented. The locations of GFUM and DYNC GPS stations which are discussed in the main article are marked as yellow triangles. Inset: map of Iceland (modified after ref.^17^) outlining its volcanic zones (e.g. West Volcanic Zone (WVZ), East Volcanic Zone (EVZ), North Volcanic Zone (NVZ)) and transform zones (e.g. South Iceland Seismic Zone (SISZ) and Tjornes Fracture Zone (TFZ)). The Reykjanes Ridge and Reykjanes Peninsula Rift (RPR), and the Kolbeinsey Ridge which mark the limits of the volcanic zone are illustrated for reference. The rate of the plate spreading is 9.8 mm yr^−1 ^^17,47^. The shaded gray area is the region covered by the Landsat-8 image in the main figure.

Bárðarbunga and Grímsvötn are two subglacial basaltic volcanoes located $\sim 27$ km apart beneath the Vatnajökull ice cap. They are isotopically distinct systems^13^ and are both sitting above the center of the mantle plume in Iceland^14^ (Fig. 1). Grímsvötn volcano hosts a 10–12 km wide and 200–300 m deep caldera complex. Geodetic measurements from its last eruption reveal a 1.7 km-deep shallow magma chamber^15^. Although, a low seismic velocity anomaly at around 3–4 km depth has been previously observed and identified as a magma chamber along with a deeper dense body inferred from gravity measurements^15,16^. Grímsvötn is Iceland’s most active volcano erupting once per decade. Its post-eruptive deformation patterns for the last three eruptions (i.e. 1998, 2004 and 2011) are very similar and suggest a plumbing system characterized by at least two connected magma reservoirs^17^ beneath the volcano. In October 1996, a subglacial eruption termed as the Gjálp eruption^18^ occurred between Grímsvötn and Bárðarbunga volcanoes. However, contrasting geochemical and geophysical analyses have resulted in an unresolved debate on whether the eruption was fed by Bárðarbunga or Grímsvötn^13,19–21^. Bárðarbunga volcano has an 11-by-18 km wide and 500–700 m deep elliptic caldera^22^ with an associated fissure swarm extending 115 km to the southwest and 55 km north-northeast^22,23^. Activity at Bárðarbunga over the last 2000 years consists of (i) subglacial or (ii) major effusive fissure eruptions^23^. In August 2014, an eruptive fissure called Holuhraun (Fig. 1) has been reactivated between the Bárðarbunga and Askja volcanic systems^23^. The activity began with intense shallow seismicity that originated from Bárðarbunga and migrated toward Askja during the weeks that followed^22^. The magmatic dyke was fed by a reservoir located at $\sim 12$ km depth beneath Bárðarbunga caldera^22^. It propagated at a distance of $\sim 41$ km before it breached the surface resulting to the Holuhraun fissure eruption. The effusive eruption lasted for 6 months and produced 1.5 ± 0.2 km^3^ of lava, making the Holuhraun eruption as the largest eruption in Iceland since the 1783–1784 Laki eruption^22^.

**Figure 2:**
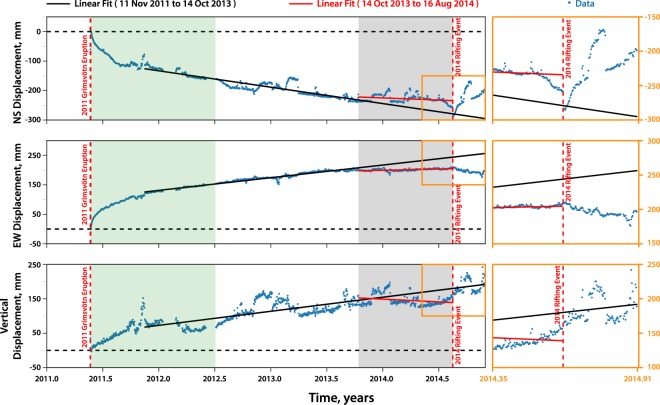
GPS time series of GFUM station from 22 May 2011 to 30 Nov 2014. The actual data are in blue points, the red solid line is the linear fit of the points within the shaded gray area (assumed shift from linear to constant trend), and the black solid line represents the linear fit prior to the shaded gray area. The latter was extended up to the end of the dataset to estimate the expected displacements after the assumed change of slope (14 October 2013). The red broken lines mark the onset of the May 2011 eruption and the August 2014 rifting event at Grímsvötn and Bárðarbunga, respectively. The horizontal black broken line is the zero-displacement reference. The shaded green area covers the dataset used during the inversion (step 1 of our approach). The insets (orange box) provide a closer look on the data points near the time of the rifting episode. Note that the vertical displacement is not corrected for GIA and seasonal effects. We applied a tectonic correction for the NS and EW components following the estimations of ref.^17^.

Between October 2013 and August 2014, NS and EW surface displacement patterns observed at GFUM— the sole GPS station in Grímsvötn located on Mount Grímsfjall (Fig. 1)— shifted from a positive linear to a nearly-constant trend (Fig. 2). Such a change of slope has not been observed during the previous post-eruptive displacement time series (Figure S1). The GPS data thus clearly shows a significant change in behavior compared to the regular trend continuously recorded over the last 10 years (i.e. 1.5 eruptive cycle) which occurred $\sim 10$ months before the 2014 major rifting event.

To quantify the displacement change, we apply a simple linear regression technique using only the linear part of the dataset prior to the assumed change in behavior (i.e. November 2011 to October 2013) and then we use the resulting slope to estimate the expected displacements at the time of the rifting (i.e. 4 UTC, 16 August 2014 is marked as the start of the major rifting event^22^). We obtained significant discrepancies of up to −39 mm and 45 mm for the EW and NS directions, respectively (see Fig. 2 and Table S1). An inflating reservoir beneath Bárðarbunga could not explain such discrepancies since displacement contributions toward the south and the east directions are expected instead (see the model displacements expected at GFUM GPS station in Table S2). Moreover, we find no similar trend variation at neighboring GPS stations close to Bárðarbunga volcano, in particular, the DYNC station which is $\sim 22$ km away from Bárðarbunga (Figure S2 and Table S3). These arguments imply that the sudden change of behavior at GFUM, one year prior to the 2014–2015 Bárðarbunga-Holuhraun eruptive activity, is most likely not directly induced by Bárðarbunga’s plumbing system. Furthermore, the ratio between the vertical and radial displacements measured at Grímsvötn remains constant through time (see Figure S3) indicating that the location of the source which induced the surface displacement has not changed through time. Given these observations, we conclude that the change of slope observed in the radial component should rather be explained by some transient process affecting Grímsvötn’s shallow reservoir. One of which is a possible variation in the magma supply rate feeding Grímsvötn’s deep reservoir.

## Model, inversion and data assimilation

**Figure 3:**
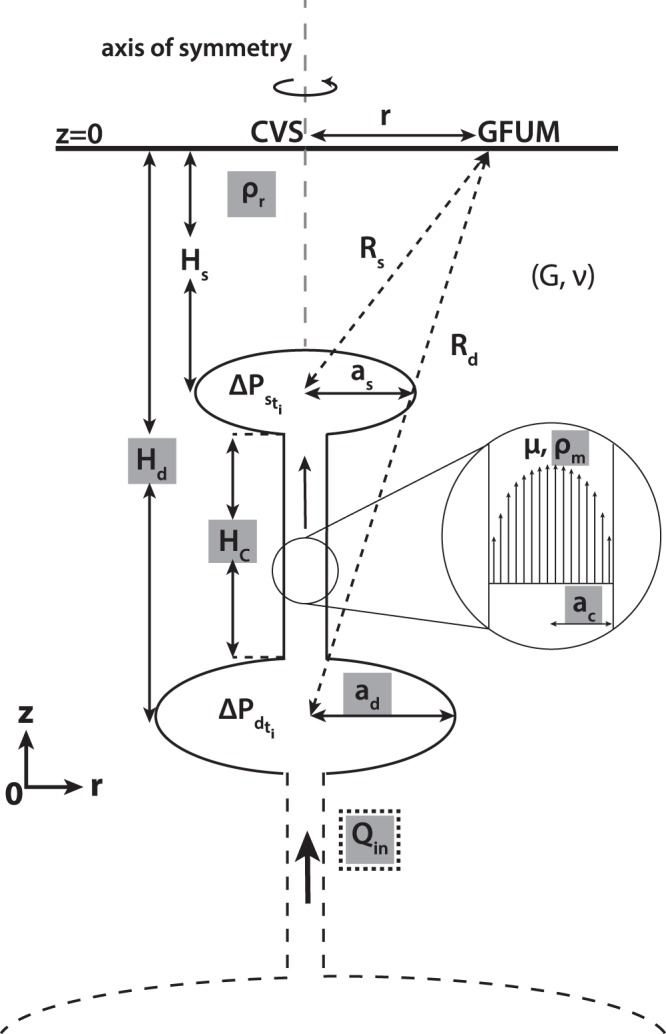
Schematic sketch of the two-chamber model, modified after ref.^12,17^. The uncertain model parameters in this study are highlighted in gray. Except for the bottom magma inflow rate, *Q*_*in*_, which is bounded by a dotted box, the rest are considered as non-evolving uncertain parameters. The GFUM GPS station, with a distance *r* from the center of the volcanic system, *CVS*, records the displacement induced by the two reservoirs. ${R}_{s}=\sqrt{{r}^{2}+{{H}_{s}}^{2}}$ and ${R}_{d}=\sqrt{{r}^{2}+{{H}_{d}}^{2}}$ are the distances of the shallow and deep reservoirs from GFUM station, respectively.

We utilize the two-magma reservoir model of ref.^17^ (Fig. 3) which represents the source of ground deformation at Grímsvötn volcano. It allows us to follow the evolution of the overpressures within the two magma reservoirs (see Methods:Model). The dynamical model is based on simple reservoir systems embedded in a homogeneous elastic crust and incompressible magma. It is consistent with the temporal evolution of the post-eruptive surface displacement at Grímsvötn, namely, an exponential trend followed by a linear one. In the case of Grímsvötn, we identified six uncertain parameters of the model (see Table 1 for the description), wherein geometrical parameters as well as parameters related to magma properties are expected to remain unchanged in one eruption cycle. Hence, we assume that all the uncertain model parameters are constant except for the basal magma inflow, *Q*_*in*_, which has a tendency to vary in time.

**Table 1:** Best-fit values of the uncertain model parameters obtained from the MCMC posterior distribution (Fig. 4). Except for *Q*_*in*_, all the other parameters are considered constant in time and are fixed to their values during data assimilation. Estimates from previous works are also presented. (Fixed parameters are: E = 25 GPa, *ν* = 0.25, *H*_*s*_ = 1.7 km, *a*_*s*_ = 2 km).

Parameters	Prior Condition	Estimated Values	
		This study	Prev. Studies
**Geometry**			
*a*_*d*_ (km), radius of the deep reservoir.	${\mathscr{N}}\mathrm{(2.2,2.52,[2.0,6.0])}$	2.39	<10^17^
*H*_*d*_ (km), depth of the deep reservoir.	*U(10, 40)*	20.56	10–35^17^; 10–20^25^
**Physics**			
Δ*ρ* = *ρ*_*r*_ − *ρ*_*m*_ (kg m^−3^), density contrast.	*U*(100, 300)	107.66	
*C* (m^3^ MPa^−1^ s^−1^), characteristic of the hydraulic connection, $C=\frac{{a}_{c}^{4}}{\mu {H}_{c}}$, where *a*_*c*_ is the radius of the conduit, *μ* is the viscosity and *H*_*c*_ = *H*_*d*_ − *H*_*s*_ is the height of the hydraulic connection.	*U(0.01, 2.35)*	0.99	
**Basal condition**			
*Q*_*in*_ (km^3^ yr^−1^), deep magma inflow rate.	*U*(0.0, 0.19)	0.046	0.01–0.05^17^
**Initial condition**			
${\rm{\Delta }}{P}_{{d}_{{t}_{0}}}$ (MPa), initial value of the overpressure in the deep reservoir.	*U*(0, 20)	4.12	

The model can be expressed in its analytical solution and differential form, which is convenient to implement for both the inversion and data assimilation. Note that for the inversion and data assimilation, only the radial component of the 2011 post-eruptive dataset is exploited due to uncertain glacial isostatic adjustment (GIA) contribution and low accuracy of the vertical component at GFUM station^17^.

To ensure that the evolution of *Q*_*in*_ can be tracked with only using the radial component of one GPS station, we first perform a synthetic test following the EnKF approach of ref.^12^. Successful results are obtained provided that non-evolving uncertain model parameters are well-estimated and fixed prior to data assimilation (Figure S4).

Given the results of the synthetic case, we then develop a two-step approach suitable for our problem and to follow the behaviour of *Q*_*in*_ (see Figure S5 for the summary and Methods:Bayesian-based Inversion and Data Assimilation for details). Step-1: We apply a Bayesian-based inversion through the Markov Chain Monte Carlo (MCMC) algorithm to first constrain the non-evolving uncertain model parameters and obtain a prior distribution for *Q*_*in*_ using only the initial part of the 2011 post-eruptive dataset. Step-2: We then implement the Ensemble Kalman Filter (EnKF)^24^ as a data assimilation technique, following the strategy developed by ref.^12^ by sequentially assimilating the 2011 post-eruptive radial data until before the rifting event.

## Results

**Figure 4:**
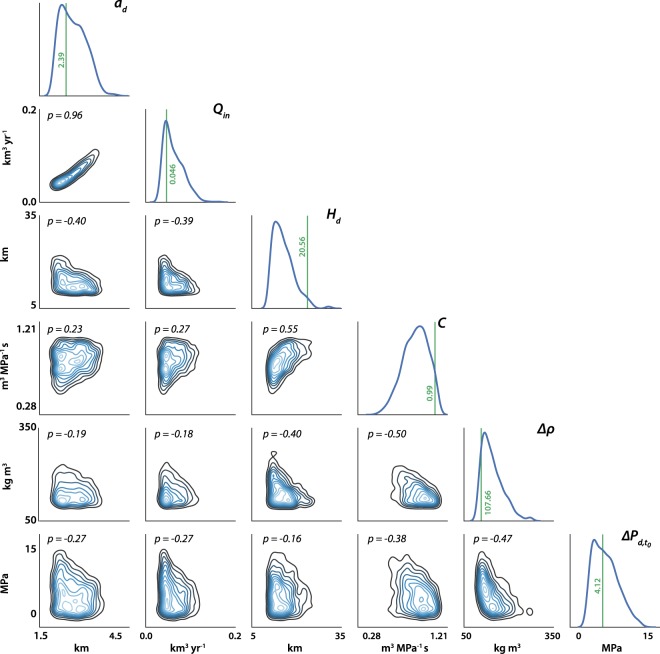
Posterior probability density functions (PDF) of the uncertain model parameters after MCMC inversion (step 1). The marginal PDF for each uncertain parameter is shown in the diagonal histogram plots. The green vertical lines with numbers indicate the best-fit values of the parameters. The off-diagonal contour plots are the joint kernel-density estimate between pairs of parameters with their corresponding Pearson correlation coefficients. A p-value close to ±1 implies strong correlation between the parameters.

The best-fit values of the uncertain model parameters after step-1 are marked as green lines in Fig. 4 and are also summarized in Table 1. Despite the non-uniqueness, these values are consistent with the data, the physics of the model and the results of previous studies^15,17,25^, such that we are able to fix the non-evolving parameters and proceed to step-2 to follow the variation of *Q*_*in*_.

**Figure 5:**
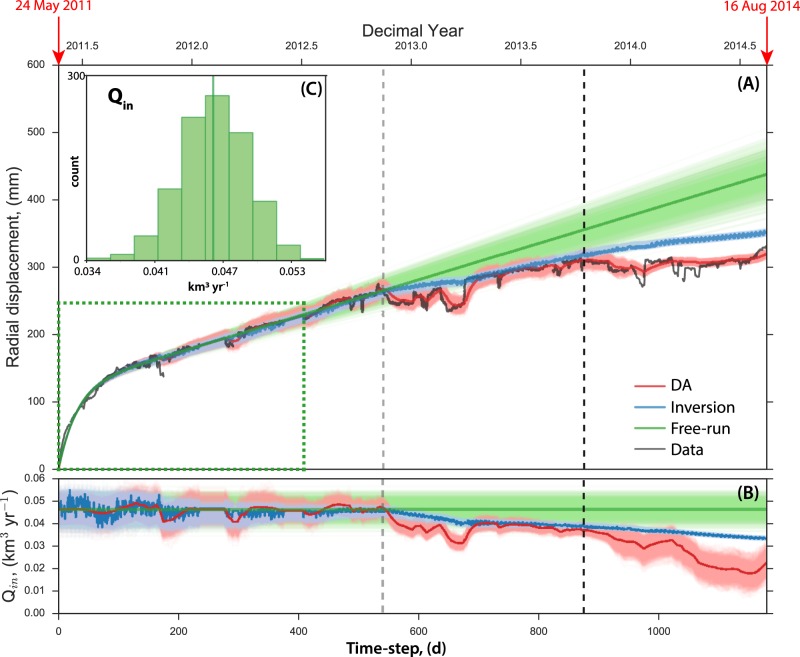
Data-fits and *Q*_*in*_ estimates. (**A**) The entire 2011 post-eruptive dataset used in this study (black) and the resulting data-fits by: (1) solely free-running the dynamical model (green), (2) performing MCMC based on a classical inversion approach/setup (blue), and (3) data assimilation via EnKF (red). The green dotted box covers the dataset used to estimate the non-evolving uncertain parameters (step 1). The robustness of each approach is depicted on how it fits the radial displacement dataset which clearly favors the EnKF method. (**B**) Estimated value of the magma inflow rate, *Q*_*in*_, as a function of time using: the free-run (green), EnKF (red) and MCMC (blue). Note that the gray and black broken lines (A and B) correspond to the points where a decreasing trend in *Q*_*in*_, tracked via EnKF, are observed. (**C**) The distribution of *Q*_*in*_ used as a prior information for the free-run, the data assimilation and the inversion.

If no observation is used to correct the dynamical model, the result is called the “Free-run” (Fig. 5) where the model is only propagated forward in time. Obviously, if the model is almost a perfect representation of the observations wherein the model parameters are well-constrained and remain constant in time as in the case of the initial part of our dataset, we expect to have a good fit with the radial dataset. Note that although we only use an early subset of the radial displacement data for the inversion (i.e. dotted green box in Fig. 5A), the inferred best-fit values of the uncertain model parameters are able to match the radial component of the dataset up to *t*_*step*_ = 545 d and appear to be consistent with the vertical component (Figure S6). It is unclear though if *t*_*step*_ = 545 d marks an episode of a true decrease in magma inflow rate or is just a part of some transient noise that affected the dataset. The latter case would require a lower value of *Q*_*in*_ at *t*_*step*_ = 0 to fit the time series up to our assumed change of slope using the forward model.

The assimilation of radial displacement using the strategy that we have developed not only results to a robust fit to the entire dataset but also enables us to follow closely the decreasing trend of *Q*_*in*_ (Fig. 5). We obtain a minimum rate of 0.007 km^3^ yr^−1^ from the ensemble of *Q*_*in*_ estimates which corresponds to a drop of 0.039 km^3^ yr^−1^ (85% decrease) relative to its prior value. To ensure that the decrease in *Q*_*in*_ is a true episode after our assumed change of slope and is not influenced by the set of values that we fixed, we run two independent cases of data assimilation (see Supplementary Material). Results show that regardless of the set of non-evolving uncertain parameters and prior distribution of *Q*_*in*_, the sudden drop in the magma inflow rate is evident for all the cases after the assumed change of slope (Figure S7).

Another interesting result is that if we follow the similar approach to track *Q*_*in*_ by first fixing non-evolving uncertain parameters, but use an inversion approach (i.e. MCMC) as a second step instead of data assimilation, we find that MCMC slightly detected the change in *Q*_*in*_, however did not yield a strong satisfactory fit with the data (Fig. 5). The main difference comes from the fact that with MCMC, we invert all the observations that are previously acquired at each given time step considering an effective constant value of *Q*_*in*_ over the whole period (i.e. using the integral analytic formula). Whereas with the assimilation strategy, we apply the differential equation between each time step such considering an evolving magma supply rate, *Q*_*in*_.

Furthermore, note that in classic inversion setup, the model used to interpret the data is assumed “perfect”. In such case, the source of error is often only attributed to the data, either to perturbations in the acquisitions, or to instrument noise, or to data pre-processing or to the sum of these errors. However, in reality, models are embedded with noise and are oversimplified representations of the complex system that we observe. Ref.^12^ illustrated through synthetic cases that if the dynamical model used explains the observed data well and that there is no transient change in the uncertain model parameters (i.e. they are constant over time), then both data assimilation using EnKF and inversion via MCMC can track the state variables (e.g. overpressures within the reservoirs) and also estimate the true values of the uncertain model parameters (e.g. basal magma inflow rate and radius of the deep reservoir).

However, if the uncertain model parameter varies over time, such as the case of *Q*_*in*_ in this study, then EnKF will be more appropriate. In EnKF, the model error covariance, $P=\overline{(X-\overline{X})(X-\overline{X}{)}^{T}}$, computed from a large number of perturbations of uncertain model parameters (*Q*_*in*_ for example) is an approximation of the real model error. In practice, we use a large ensemble of models in order to best represent the model error. Furthermore, by using a multiplicative inflation (see Methods:Model) which is a tuning step in data assimilation, underestimated model errors related to EnKF process and/or unaccounted source of model error are compensated.

## Implications of the change in magma supply rate at Grímsvötn

The estimated decrease of the magma supply rate measured at Grímsvötn corresponds to a mimimum deficit of 0.016 km^3^ of magma for the Grímsvötn plumbing system when during the same period, magma accumulation is expected at Bárðarbunga^22^. Note that the missing volume of magma was calculated by integrating the inverted curve of EnKF-derived *Q*_*in*_ in Fig. 5B. The volume emplaced beneath Bárðarbunga during the same period cannot be quantified due to the lack of geodetic data (the closest GPS point is DYNC located at more than 20 km). A similar anti-correlated behavior has been previously observed between isotopically different volcanic systems in Hawaii, namely, Kilauea and Mauna Loa volcanoes, based on their eruptive activities^26^. It was then interpreted as due to stress transfer over the 35 km distance separating the two systems through pore-pressure variations in a thin asthenospheric melt accumulation layer. Evidences from geodetic observations for connections between volcanic systems have been noted before, but only for systems spaced less than 10 km apart^7^. Mechanisms invoked to explain nearby volcanic systems interactions include stress changes, lateral hydraulic connections and a common asthenospheric magma supply as for the Hawaiian case. In the Grímsvötn-Bárðarbunga case, we can exclude the stress change effect due to the large distance separating the two systems^7^.

**Figure 6:**
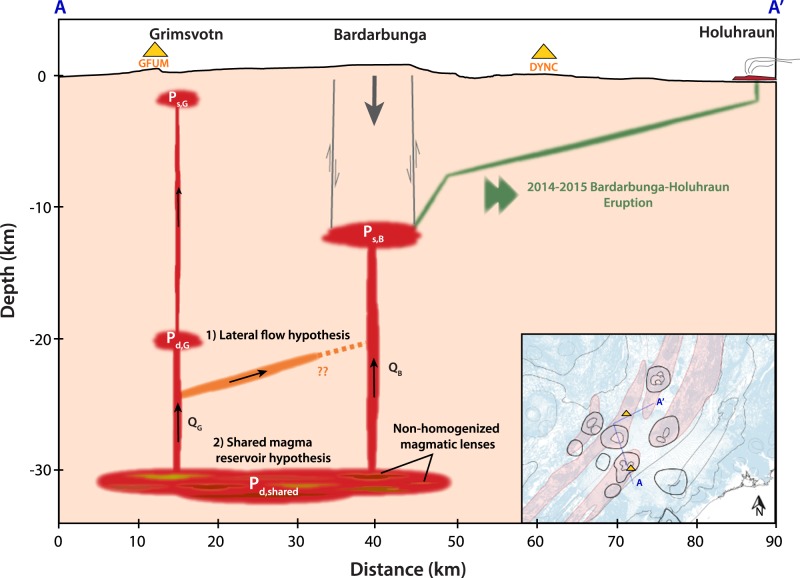
Proposed schematic cross-section beneath Grímsvötn and Bárðarbunga illustrating the two possible deep mechanisms connecting the two volcanic systems: (1) lateral flow hypothesis and (2) shared magma reservoir hypothesis. GFUM and DYNC GPS stations are represented as yellow triangles. The link between Bárðarbunga and Holuhraun during the 2014–2015 eruption (green sketch) is shown after ref.^22^; however it does not follow the cross-section path in the inset figure.

Two scenarios possibly caused the decrease in magma supply to Grímsvötn between October 2013 and August 2014 **(**Fig. 6**)**: (1) the magma that feeds Grímsvötn’s mid-crustal reservoir at $\sim 20$ km depth was transported toward Bárðarbunga’s volcanic system through an existing deep fracture (i.e. lateral flow hypothesis), and (2) there was a drop in the relative pressure difference between Grímsvötn’s mid-crustal reservoir and a much deeper reservoir shared by Bárðarbunga and Grímsvötn at more than 20 km depth (i.e. shared magma reservoir hypothesis).

For the first case, the minimum volume of magma that leaked out of Grímsvötn is 0.016 km^3^, assuming that the magma is an incompressible fluid. This assumption in our modeling approach may not be fully accurate because in reality, magma is a compressible fluid containing exsolved volatiles at shallow depth. Significant compressibility would result in much larger erupted volumes (i.e. 4 to 5 fold higher)^2,27^, implying that the “missing” amount of magma may be as much as 0.08 km^3^. On one hand, several studies claimed that the true volume change of a penny-shaped sill is not significantly affected by magma compressibility^27,28^. On the other hand, ref.^15^ noted the important role of magma compressibility at Grímsvötn (i.e. the volume of the 2011 erupted magma is 10 times of the geodetically-derived chamber volume assuming a spherical-shaped chamber), however, its effect to the calculated magma supply rate is of second order^2^. The lack of spatial information in our dataset may also contribute to underestimating the missing magma volume since there is only one GPS time series that we can exploit at Grímsvötn. Indeed, our synthetic case shows the ability of EnKF to detect the sudden change in *Q*_*in*_ given a spatially-limited dataset, whilst the convergence to the true value may require some time (Figure S4).

As to whether or not the “missing” magma may have reached the Bárðarbunga’s magmatic system is yet to be addressed. While the volume of “missing” magma is only ∼3–20% of the estimated 2014 dyke intrusion^22,23^, it could be enough to trigger a magmatic reservoir rupture beneath Bárðarbunga and initiate magma propagation from the storage zone. It is possible, assuming that the magma reached Bárðarbunga’s magmatic system and provided that its storage zone was already in a pressurized stage before this additional magma inflow. Lateral dyke propagation between Grímsvötn and Bárðarbunga volcanoes for the 1996 Gjalp eruption has been previously proposed^19^, which was further supported by a mechanical model indicating that dyke sharing between the two volcanoes is possible^29^. Recent stress field model around Bárðarbunga from the 2014–2015 Holuhraun rifting event also corroborates the lateral flow hypothesis, suggesting that intrusions may have occurred in the past releasing part of the stress caused by plate spreading but were undetected due to their subglacial nature^30^. Although, if the magma laterally migrated away from Grímsvötn but was arrested at depth, the induced stress change can only be very localized. High resolution geophysical imaging of the northwest Vatnajökull region may provide further clues and deeper insights in the future about the shallow crustal structure beneath the icecap.

One significant argument against the lateral flow hypothesis is that no increase of deep seismicity was recorded during the 10 months preceding the rifting event. Deep seismicity rather indicates a continuous vertical rise of magma 12 km to the south-east of Bárðarbunga caldera^31^. Another one is that the magma emitted during the Holuhraun eruption is compositionaly similar to the Bárðarbunga volcanic system^32^.

For the second case, assuming that the ascent of magma from a deep source to an upper magma chamber (Fig. 6) follows a Poiseuille flow^33^, a drop in the pressure between the common reservoir of Grímsvötn and Bárðarbunga (*P*_*d*,*shared*_) and the mid-crustal reservoir of Grímsvötn (*P*_*d*,*G*_) will decrease the magma inflow rate toward the latter (*Q*_*G*_). The sudden pressure drop is due to the withdrawal of a large amount of magma in the shared reservoir (*P*_*d*,*shared*_) that may have been caused by the activation or reactivation of a connection between Bárðarbunga (*P*_*s*,*B*_) and the shared (*P*_*d*,*shared*_) reservoirs. Ref.^34^ estimated the mantle-crust boundary in Iceland to be at $\sim 30$ km depth. This mantle-crust boundary is a suitable place for the accumulation of melt which can be the source of magma for both Grímsvötn^25^ and Bárðarbunga’s magmatic systems^35^. As Grímsvötn and Bárðarbunga basalts have distinct isotopic signatures, the term of “shared reservoir” has to be considered as a magmatic domain similar to a porous melt accumulation layer^26^. Each melt-filled pore space then shares a mechanical connection controlled by pore pressure gradient for instance. In this case, the shared reservoir can contain different and non-homogenized magmatic lenses because the stress transfer can be significantly faster than the flow of melt within the porous zone^26^. Here, the lack of information regarding the temporal pressure evolution in Bárðarbunga’s storage zone before the rifting event prevents us to further model the connection, such that we cannot constrain the porous layer thickness or diffusivity.

A connection of sorts between Grímsvötn and Bárðarbunga was previously identified by ref.^36^. The authors show that the volcanic activity in the northwest Vatnajökull region since A.D. 1200 is periodic (i.e between 130–140 yr) with low activity lasting 50–80 yr followed by high activity of similar duration. In all peaks in activity except for one, the eruptions occur at Grímsvötn. During the initial part of the 1700s, Bárðarbunga was observed to be responsible for the unrest while at the same time, Grímsvötn was relatively quiet. This argument strongly favors the shared magma reservoir hypothesis, that is, the two volcanoes are connected at depth by a common reservoir controlled by dynamic stress transfer. In addition, petrological and geochemical analyses from Grímsvötn’s eruptive products and Holuhraun’s 2014–2015 lava all indicate magma recharge from depth^25,32^.

Whilst we propose two possible mechanisms that connect Grímsvötn and Bárðarbunga’s magmatic systems, the aforementioned arguments are much in favor of the shared magma reservoir hypothesis and provide sufficient evidences to conclude that magma flowed beneath Bárðarbunga starting at least 10 months before the initiation of the rifting event. We consequently argue that a strong interplay between the surge in magma supply in an already-pressurized source and the rifting episode triggered the rupture of Bárðarbunga’s magma chamber, rather than the reduction of the minimum principal compressive stress linked to the rifting alone as proposed by ref.^37^. Bárðarbunga’s location, being on top of Iceland’s mantle plume and in a rift zone, makes it a suitable place for the interplay. Subsequently, the withdrawal of magma and the gradual collapse of the caldera are both responsible for sustaining the eruption for up to $\sim 6$ months^22^.

## Lessons learned

Although geodetic observations have greatly improved over the last decades, our ability to infer the characteristics of the deep systems underlying volcanoes remains a great challenge. On one hand, we do not have a direct observation of the plumbing system beneath volcanoes such that we only infer their characteristics based on ground surface measurements. Thus, only an oversimplified representation of their complex nature is possible. On the other hand, model-data fusion techniques applied to inverse problems in volcanology have remained under the assumption of a “perfect” model therefore largely ignoring errors related to the representation of the dynamics of the real system. In the case of estimating a parameter that varies through time, knowledge about the parameter’s true behavior is incorrect as we have shown previously when classical static inversion was used to track time-varying *Q*_*in*_.

The advantage of using sequential data assimilation over a classic bayesian-based inversion method to follow the evolution of the deep magma supply rate is clear and represents a first successful application of EnKF in volcanology. Our framework is simple, but it help us better understand subsurface processes occurring between magmatic reservoirs (e.g. Grímsvötn and Bárðarbunga). We thus demonstrate that in addition to the interest of predicting volcanic eruptions^12^, sequential assimilation of geodetic data based on a dynamical model has a real and unique potential to give insights into the deep plumbing system of volcanoes and evolution of bottom conditions though time.

The sudden decrease of magma supply to Grímsvötn between 2013 and 2014 was a transient phenomenon caused by the accumulation of magma beneath the Bárðarbunga’s reservoir. The absence of a similar event based on previous post-eruptive displacement patterns at Grímsvötn suggests that the observed shift is an unusual event. After the 2014 rifting episode, radial displacement pattern at GFUM GPS station began increasing again, following a positive linear trend (Figure S1). However, it is difficult to analyze the displacements after August 2014 due to the combined contributions of viscoelastic relaxation caused by the rifting, the migration of the dyke and the volcanic deformation related to Grímsvötn’s activity alone.

Our analyses suggest a strong interplay between a surge in magma supply at Bárðarbunga and the rifting episode that triggered the 2014–2015 eruptive activity which was subsequently followed by gradual caldera collapse resulting in the $\sim 6$-month long eruption. This transient reduction of magma supply rate at Grímsvötn may have postponed the next eruption, thereby increasing the duration of the inter-eruptive period.

## Methods

### Model

The volcano model used in this work is based on the two-chamber model published by ref.^17^ with a slight modification to the original equations to account for initial overpressure values:

1 $$\frac{{\rm{\Delta }}{P}_{{s}_{{t}_{i+1}}}-{\rm{\Delta }}{P}_{{s}_{{t}_{i}}}}{{t}_{i+1}-{t}_{i}}=\frac{3\pi E{{a}_{c}}^{4}}{\mathrm{128(1}-{\nu }^{2})\mu {H}_{c}{{a}_{s}}^{3}}((({\rho }_{r}-{\rho }_{m})g{H}_{c}+({\rm{\Delta }}{P}_{{d}_{{t}_{0}}}-{\rm{\Delta }}{P}_{{s}_{{t}_{0}}}))+{\rm{\Delta }}{P}_{{d}_{{t}_{i}}}-{\rm{\Delta }}{P}_{{s}_{{t}_{i}}})$$

2 $$\frac{{\rm{\Delta }}{P}_{{d}_{{t}_{i+1}}}-{\rm{\Delta }}{P}_{{d}_{{t}_{i}}}}{{t}_{i+1}-{t}_{i}}=\frac{3E}{\mathrm{16(1}-{\nu }^{2}){{a}_{d}}^{3}}{Q}_{in}-\frac{{{a}_{s}}^{3}}{{{a}_{d}}^{3}}\frac{{\rm{\Delta }}{P}_{{s}_{{t}_{i+1}}}-{\rm{\Delta }}{P}_{{s}_{{t}_{i}}}}{{t}_{i+1}-{t}_{i}}$$

here, the two-magma chamber model only represents the upper part of a multiple-reservoir system. Given a set of known but uncertain model parameters (Fig. 3), the forward model computes the evolution of the overpressures in the shallow and deep reservoirs which are directly related to the deformation measured at the surface. We assume the following for the known parameters of our model: the shapes of the reservoirs are sills, the Young’s modulus, *E* = 25 GPa, the depth and the radius of the shallow reservoir are *H*_*s*_ = 1.7 km and *a*_*s*_ = 2.0 km^15^, respectively. The uncertain model parameters are classified into two: (a) constant/non-evolving (i.e. all uncertain parameters except *Q*_*in*_) and (b) time-varying (i.e. *Q*_*in*_).

Note that any dynamical model can be used for data assimilation, however, the interpretation we made clearly depends on our model choice. We used the two-reservoir model because of its ability to well explain the temporal evolution of the displacement recorded at Grímsvötn (i.e. exponential followed by a linear trend) after each erupted event. Other models considering only one reservoir fed by a deep and constant pressure source could potentially explain the same temporal evolution by introducing either a complexity in the encasing medium rheology (i.e. damage, viscoelastic behavior^38,39^) and/or in the magma properties (i.e. crystallisation, degassing, compressibility^39,40^). However, there is a strong geochemical evidence for probably at least several deep reservoirs beneath the Grímsvötn system^25^. In particular, Grímsvötn lavas that are averaged from previous eruptions have a crystallisation depth of 15 ± 5 km^25^ — a value consistent with the result of our inversion. Besides, the deep magma supply rate is expected to fluctuate through time^1–3^. The facts that (1) such fluctuation is not observed during the previous eruptive cycle, (2) it seems to be transient and (3) it occurs simultaneously with a rifting event mobilizing a large volume of magma in a close volcanic system, are strong arguments in favor of the model preferred in this study. Having GPS data at a distance of 15 km from Grímsvötn could confirm this effect of the deep reservoir (see Figure S8).

### Data

Data are analyzed using the Bernese 5.2 software^41^, with absolute antenna phase center offset models, together with precise orbits, earth rotation parameters, ocean tidal loading and atmospheric tidal loading estimates. Velocities and time series were estimated in the ITRF2014 reference frame^41^ with discontinuities associated with this reference frame and expressed in terms of the plate boundary reference frame^17^. We followed the resolution strategy with (1) an initial ionosphere-free analysis with calculation of the residuals; (2) a residual analysis; (3) code-based wide-lane ambiguity resolution for all baselines^42^, using differential code bias (DCB) files when available and calculation of the ionosphere-free solution with the introduction of resolved Melbourne-Wübbena linear combination ambiguities; (4) phase-based wide-lane (L5) ambiguity resolution for baselines <200 km and computation of the ionosphere-free solution with the introduction of resolved ambiguities; (5) resolution of the previously unresolved ambiguities for baselines <2000 km using the quasi ionosphere-free strategy of resolution; (6) direct L1/L2 ambiguity resolution for baselines <20 km with the introduction of an ionosphere model; (7) calculation of the normal equations; (8) a compatibility test between the daily free solution and ITRF2014 solution, selection of compatible ITRF2014 stations and (9) transformation of the daily normal equation in the ITRF2014 reference frame with a six-parameter Helmert solution (three translation parameters and three rotation parameters) using the ITRF2014 selected stations (BARH, BOGO, EPRT, ESCU, HERT, HLFX, KARL, KHAR, NEWL, REDU, RIGA, SASS, SCH2, SHE2, SKE0, STAS, THU2, THU3, TRDS, VARS, VOL0). During these steps, site-specific troposphere parameters are estimated every two hours. Normal equations are analyzed together to determine accurate velocities in the ITRF2014 reference frame with the introduction of ITRF2014 coordinates and velocities. For time series of stations supposed to be linear (not GFUM), outliers and new discontinuities were detected using the “Find Outliers and Discontinuities in Time Series” tool in the Bernese 5.2 software that reduces, step-by-step, the discrepancy between the functional model and the time series due to statistical adjustment^43^, taking annual and seasonal fluctuations into account. As the Bernese 5.2 software underestimates the daily errors given that systematic errors or mismodeled parameters are not included in the formal error^41^, we rescaled the formal errors by multiplying them by a factor of 10 to obtain a more realistic estimated error.

Note that the tectonic trend at GFUM GPS station is removed with slopes of −2.7 mm yr^−1^ and 7.5 mm yr^−1^ for the NS and EW components, respectively^17^.

### Bayesian-based inversion

To constrain the uncertain parameters of our model that remain constant in time (e.g. ${a}_{d},{H}_{d},C,{\rm{\Delta }}\rho ,{\rm{\Delta }}{P}_{{d}_{{t}_{0}}}$) and to obtain a prior distribution for the evolving one (*Q*_*in*_) before data assimilation, we perform a Bayesian-based inversion using the Markov Chain Monte Carlo (MCMC) algorithm. We follow the step-by-step procedure presented in Figure S5 (i.e. Step 1). In MCMC, uncertain model parameters independently drawn from given a priori distributions are constrained by accepting model predictions that better fit observations and by randomly accepting those that do not fit to avoid being trapped to a local minima^44^. In particular, we built our approach using the PyMC2 python module. The classic linear inverse problem is described as:

3 $$D=G(m)+\varepsilon $$

where *D* is the vector of observation or data, *G* is the forward model, *m* is a vector of the uncertain model parameters and *ε* is the observation error. In our case, $m=[{a}_{d},{H}_{d},C,{\rm{\Delta }}\rho ,{\rm{\Delta }}{P}_{{d}_{{t}_{0}}},{Q}_{in}]$, *D* is the 2011 post-eruptive GPS dataset (we only use the radial displacement component up to *t*_*step*_ = 409 d) and *G* is the analytical solution (obtained by combining equations 3, 11 and 12 of ref.^12^) to the forward model described in equations (1) and (2). In the Bayesian framework, the posterior probability associated with *m* is sampled based on a likelihood function, *P*(*D*|*m*), that calculates how well the data fits the model and a prior knowledge about the uncertain model parameters, *P*(*m*):

4 $$P(m|D)\propto P(D|m)P(m)$$

Note that the errors in the resulting posterior distributions are only related to the observation error and uncertainties of the prior distribution of *m*. Any error related to how the model, *G*, represents the reality is not taken into account. Such that in the case of a time-varying parameter, this approach is not the optimal strategy to use.

We first scale *m* and then we compute for the posterior distributions incorporating an Adaptive Metropolis (AM) step method. The latter is to avoid the problem of convergence due to possible “trade-offs” between the six uncertain model parameters. The AM method is a more intelligent way of fitting the parameters by block updating them using a multivariate jump distribution. We performed 2.0 × 10^5^ individual samples with each calling the forward model *G*. To make sure we converged to a good estimate and maintain no autocorrelation, half of the samples are burned and the remaining samples are thinned by a factor of 100 such that we end up by having 1000 samples. Because of the simplicity of the forward model, it only took around 30 minutes to simultaneously obtain the posterior distributions of the uncertain model parameters.

From the constructed posterior distribution, we pick the set of best-fit parameters by computing the misfit relative to the data points within the dotted green box in Fig. 5. The results are summarized in Table 1 and are marked using green vertical lines in the marginal distribution shown in Fig. 4.

We perform similar inversion procedure to obtain the two other set of priors that we tested: case (1) using the entire 2004 radial displacement dataset and case (2) adopting the values of *a*_*d*_, *H*_*d*_ and Δ*ρ* from case 1 and then re-estimating the uncertain parameters that could vary from one eruption to another (e.g. *Q*_*in*_, *C*, and ${\rm{\Delta }}{P}_{{d}_{{t}_{0}}}$).

### Data assimilation

We closely followed the strategy developed by ref.^12^ to assimilate geodetic data into a forward dynamical model. A step-by-step data assimilation strategy using EnKF is presented in Figure S5 (i.e. Step 2). The assimilation is divided into two steps: (1) the forecast step and (2) the update step (or analysis). The forecast step is the part where an N-ensemble of models (i.e. *N* = 1000) are generated using the forward dynamical model given a previous or prior distribution of the state vector, *X*:

5 $${X}_{{t}_{i+1}}^{f}= {\mathcal M} {X}_{{t}_{i}}^{a}+{q}_{{t}_{i}}$$

f and a: denote the forecast and analysis, respectively, ${\mathcal M}$: the model operator that relates the system from time *t*_*i*_ to *t*_*i*+1_ and *q*: the model error. In our case, the state vector is expressed as:

6 $$X=[\begin{array}{c}{\rm{\Delta }}{P}_{s}\\ {\rm{\Delta }}{P}_{d}\\ {Q}_{in}\end{array}]$$

and ${\mathcal M}$ is of the form:

7 $$[\begin{array}{c}{\rm{\Delta }}{P}_{{s}_{{t}_{i+1}}}\\ {\rm{\Delta }}{P}_{{d}_{{t}_{i+1}}}\\ {Q}_{i{n}_{{t}_{i+1}}}\end{array}]=[\begin{array}{ccc}1-{C}_{1}{\rm{\Delta }}t & {C}_{1}{\rm{\Delta }}t & 0\\ {C}_{1}{C}_{2}{\rm{\Delta }}t & 1-{C}_{1}{C}_{2}{\rm{\Delta }}t & 0\\ 0 & 0 & 1\end{array}][\begin{array}{c}{\rm{\Delta }}{P}_{{s}_{{t}_{i}}}\\ {\rm{\Delta }}{P}_{{d}_{{t}_{i}}}\\ {Q}_{i{n}_{{t}_{i}}}\end{array}]+[\begin{array}{c}{C}_{1}{A}_{1}{\rm{\Delta }}t\\ ({A}_{2}-{C}_{1}{C}_{2}{A}_{1}){\rm{\Delta }}t\\ 0\end{array}]$$

where $${C}_{1}=\frac{3\pi E{{a}_{c}}^{4}}{\mathrm{128(1}-{\nu }^{2})\mu {H}_{c}{{a}_{s}}^{3}}$$, $${A}_{1}=({\rho }_{r}-{\rho }_{m})g{H}_{c}+({\rm{\Delta }}{P}_{{d}_{{t}_{0}}}-{\rm{\Delta }}{P}_{{s}_{{t}_{0}}})$$, $${A}_{2}=\frac{3E{Q}_{in}}{\mathrm{16(1}-{\nu }^{2}){{a}_{d}}^{3}}$$ and $${C}_{2}=\frac{{{a}_{s}}^{3}}{{{a}_{d}}^{3}}$$.

The assimilation interval is set to Δ*t* = 1 day and we expect to have GPS data every day (i.e. frequency of observation, *f*_*obs*_ = 1). A Gaussian prior for the uncertain model parameter is required by EnKF to achieve an unbiased and optimal estimate. However, to ensure that the predicted value is within the correct physical boundaries, we redefine the distribution of *Q*_*in*_ (expressed in km^3^ yr^−1^) prior to the implementation of EnKF to a truncated Gaussian distribution with the mean centered on its best-fit value obtained from the inversion, the standard deviation is set to $\sim 0.003$ km^3^ yr^−1^ and the upper and lower limits are fixed to [*a* = 0, *b* = 0.19] (see Fig. 5C).

In the context of data assimilation, Δ*P*_*s*_ and Δ*P*_*d*_ are called state variables because they are directly linked to the observations, while *Q*_*in*_ is termed as an uncertain model parameter and is only updated by the covariance between it and the state variables during the update step. We scaled the forecast state vector, *X*^*f*^, (i.e. 10^7^ and 10^−1^ for the overpressures and *Q*_*in*_, respectively) and then we imposed an inflation factor, *ρ*_*infl*_ = 0.05 (i.e. *X*^*f*^ = (1 + *ρ*_*infl*_)*X*^*f*^, see section 4.2 of ref.^12^) if the standard deviation of *Q*_*in*_ at *t*_*i*+1_ falls below its standard deviation at *t*_0_. The latter is to prevent the ensemble from collapsing to a single value and also to help the filter to track the value of the time-varying parameter, *Q*_*in*_.

Once observation is available, *X*^*f*^ is updated by computing the Kalman Gain, *K*, (see equation 8 of ref.^12^) and then applying the update equation,

8 $${X}^{a}={X}^{f}+K(D- {\mathcal H} {X}^{f})$$

to obtain the vector of analysis, *X*^*a*^. Note that the value of *Q*_*in*_ remains unchanged if there is no observation. Since we only use the radial component of the displacement time series, we define the observation operator, $$ {\mathcal H} =[\begin{array}{cc}{\rm{\Gamma }}{D}_{s}r & {\rm{\Gamma }}{D}_{d}r\end{array}]$$, such that:

9 $${D}_{{t}_{i}}={u}_{{R}_{{t}_{i}}}=[\begin{array}{cc}{\rm{\Gamma }}{D}_{s}r & {\rm{\Gamma }}{D}_{d}r\end{array}][\begin{array}{c}{\rm{\Delta }}{P}_{{s}_{{t}_{i}}}\\ {\rm{\Delta }}{P}_{{d}_{{t}_{i}}}\end{array}]+{\varepsilon }_{{t}_{i}}$$

where ${\rm{\Gamma }}=\frac{\mathrm{8(1}-{\nu }^{2})}{\pi E}$, ${D}_{s}=\frac{{H}_{s}^{2}{a}_{s}^{3}}{{R}_{s}^{5}}$, ${D}_{d}=\frac{{H}_{d}^{2}{{a}_{d}}^{3}}{{R}_{d}^{5}}$ and *r* is the distance of GFUM GPS station from the center of the volcanic system (e.g. 3.5 ± 0.2 km). Lastly, we use an error variance, $R={\mathbb{E}}(\varepsilon {\varepsilon }^{T})=$ (0.015 m)^2^.

### Data availability

The GPS data analysed during the current study are available from the corresponding author on reasonable request.

## Electronic supplementary material

Supplementary File
