# Supplementary material for: Possible deep connection between volcanic systems evidenced by sequential assimilation of geodetic data
Source: Sci Rep. 2018 Aug 3;8:11702. doi: 10.1038/s41598-018-29811-x (PMC6076276; doi:10.1038/s41598-018-29811-x)
Supplement: Supplementary file 1 — Supplementary File [file 41598_2018_29811_MOESM1_ESM.pdf]

Supplementary Material: Possible deep connection between volcanic systems evidenced by sequential assimilation of geodetic data

Mary Grace Bato*[1], Virginie Pinel[1], Yajing Yan[2],
François Jouanne[1], Jean Vandemeulebrouck[1]
[1]Université Grenoble Alpes, Université Savoie Mont Blanc, CNRS, IRD, IFSTTAR, ISTerre, 38000 Grenoble, France
[2]Université Savoie Mont Blanc, LISTIC, 74000 Annecy, France
*m.grace.bato@gmail.com

Supplementary Background

The displacements induced by an inflating source beneath Bardarbunga

We modeled the expected displacements at GFUM and DYNC GPS stations induced by an inflating source beneath Bárðarbunga using the results of previous studies [1, 2, 3] as input parameters to a generalized point-pressure source equation:

$$\begin{bmatrix} u_Y \\ u_X \\ u_d \end{bmatrix} = \begin{bmatrix} \alpha \frac{(1-\nu)}{G} a^3 \Delta P \left(\frac{Y}{R^3} \right) \\ \alpha \frac{(1-\nu)}{G} a^3 \Delta P \left(\frac{X}{R^3} \right) \\ \alpha \frac{(1-\nu)}{G} a^3 \Delta P \left(\frac{d}{R^3} \right) \end{bmatrix} \tag{1}$$

The description of the parameters, the corresponding values and references used in this study as well as the results of the forward modeling are summarized in **Table S2**. Modeled displacements show that an inflating source beneath Bárðarbunga can explain the displacements at DYNC station (**Table S3** and **Figure S2**) but not at GFUM station (**Table S1** and **Figure 2**).

Data assimilation using synthetic GPS data

We adopted the setup of ref.[4] in generating the synthetic observations, except that in this case, we only produce the radial component of a GPS time series measured at one station (i.e. at $r = 3.5$ km). The frequency of incoming GPS observation is daily. Also, while generating the synthetic observations, we assumed that after $t_{step} = 875$ d, the value of Q_{in} suddenly drops to zero such that the synthetic displacement becomes constant afterwards (i.e. "Truth" in **Figure S4**). We considered a white Gaussian observation error similar to the one used for the GFUM dataset (i.e. $R = 0.015^2$ m). We then followed the state-parameter estimation discussed in the Methods part of the main article.

In **Figure S4**, we demonstrate how the radial component of a GPS time series at one station performs when tracking the sudden change of Q_{in} using EnKF. For the context of data assimilation experiments, we also presented the result of "Free-run" in **Figure S4**. Clearly, with EnKF we were able to detect the sudden drop in Q_{in} value, although it took time to approach zero.

Testing different sets of uncertain model parameters as prior inputs to EnKF

The set of best-fit values summarized in **Table 1** and illustrated in **Figure 4** is only one of the many solutions that could satisfy the observed displacement we used for the inversion. The non-uniqueness of the solution is a consequence of the poor spatial resolution of the dataset since we only have one

GPS station at Grímsvötn with six uncertain model parameters to infer. However, our main goal is not to find a unique solution to our inverse problem rather to obtain values that are consistent with the data, physics of the model and results of previous studies [5, 6, 7] such that we can fix the non-evolving ones and follow the variation of Q_{in}. In order to ensure that the obtained set of values has no affect on the results of the assimilation and on our interpretation about Q_{in}, we tested two other sets of values prior to implementing EnKF. In the first case, we obtain a set of values for the six uncertain model parameters using the 2004 post-eruptive radial dataset. In the second case, we assume that a_d, H_d and $\Delta\rho$ are constant from one eruption to another so we adopted their values from case 1 and then recalculated Q_{in}, C and $\Delta P_{d,t_0}$ using only the initial part of the 2011 radial displacement time series. **Figure S7** clearly shows that regardless of the set of prior values used as inputs to the EnKF scheme, a sudden drop in the magma inflow rate is evident after the observed change of slope, ~ 10 months before the rifting event.

Supplementary Figures

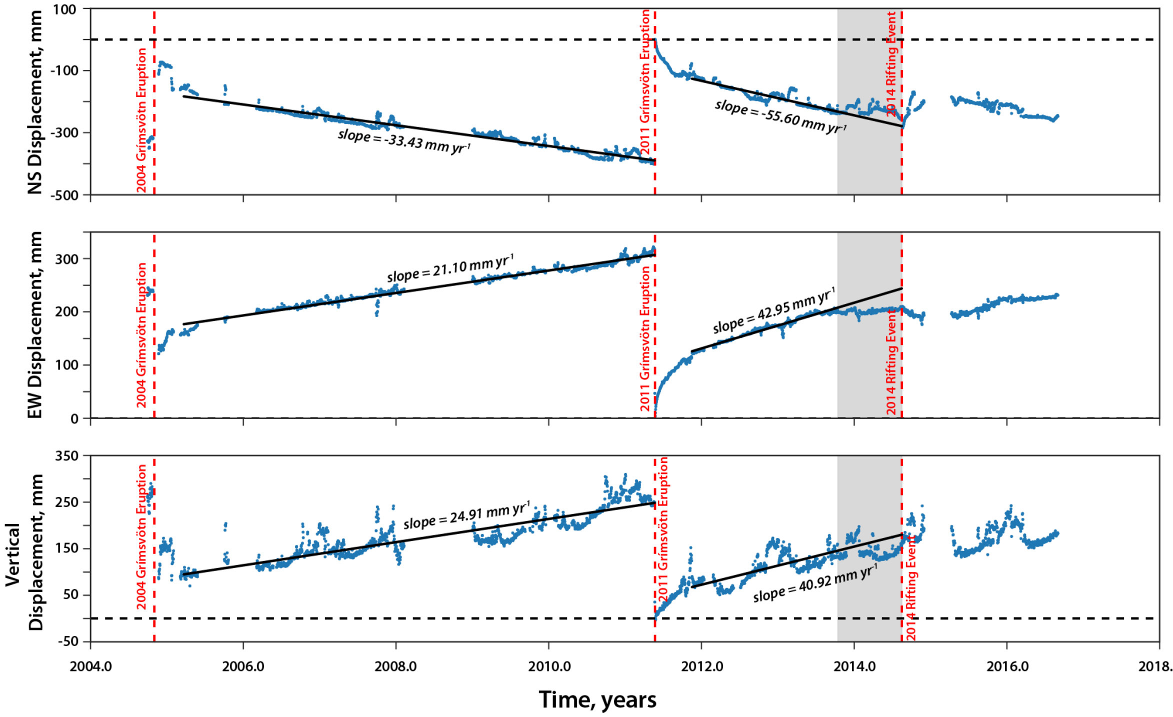

Figure S1: *GPS time series measured at GFUM station from 30 Sep 2004 to 01 Sep 2016. The blue dots are the actual data. The red broken lines mark the 2004 and 2011 Grímsvötn eruptions as well as the 2014 major rifting event. The black solid lines are the linear fit to the linear part of each post-eruptive event. The black broken lines are shown as a reference for the zero-displacement value. The shaded gray area corresponds to the assumed shift from linear to constant trend around 10 months before the 2014 rifting event. Note that the vertical component of the time series is not corrected for either GIA or seasonal effects. The horizontal component is however, corrected for tectonic trend.*

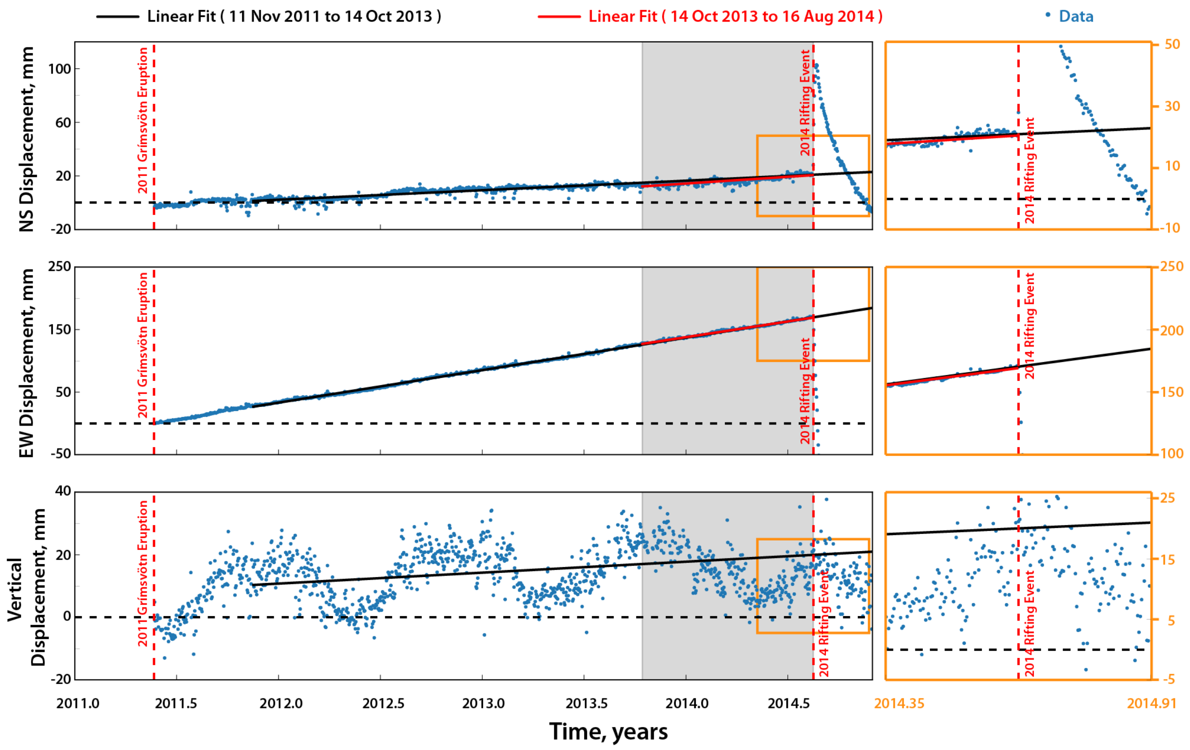

Figure S2: *GPS time series measured at DYNC station from 22 May 2011 to 30 Nov 2014. The blue dots are the actual data, the red solid line is the linear fit of the points within the shaded gray area (i.e. area that corresponds to the assumed shift from linear to constant trend detected at GFUM station), and the black solid line represents the linear fit prior to the shaded gray area. The latter was extended up to the end of the dataset. The red broken lines mark the onset of the May 2011 eruption and the August 2014 rifting event at Grímsvötn and Bárðarbunga, respectively. The horizontal black broken line is the zero-displacement reference. The insets (orange box) provide a closer look on the data points near the time of the rifting episode.*

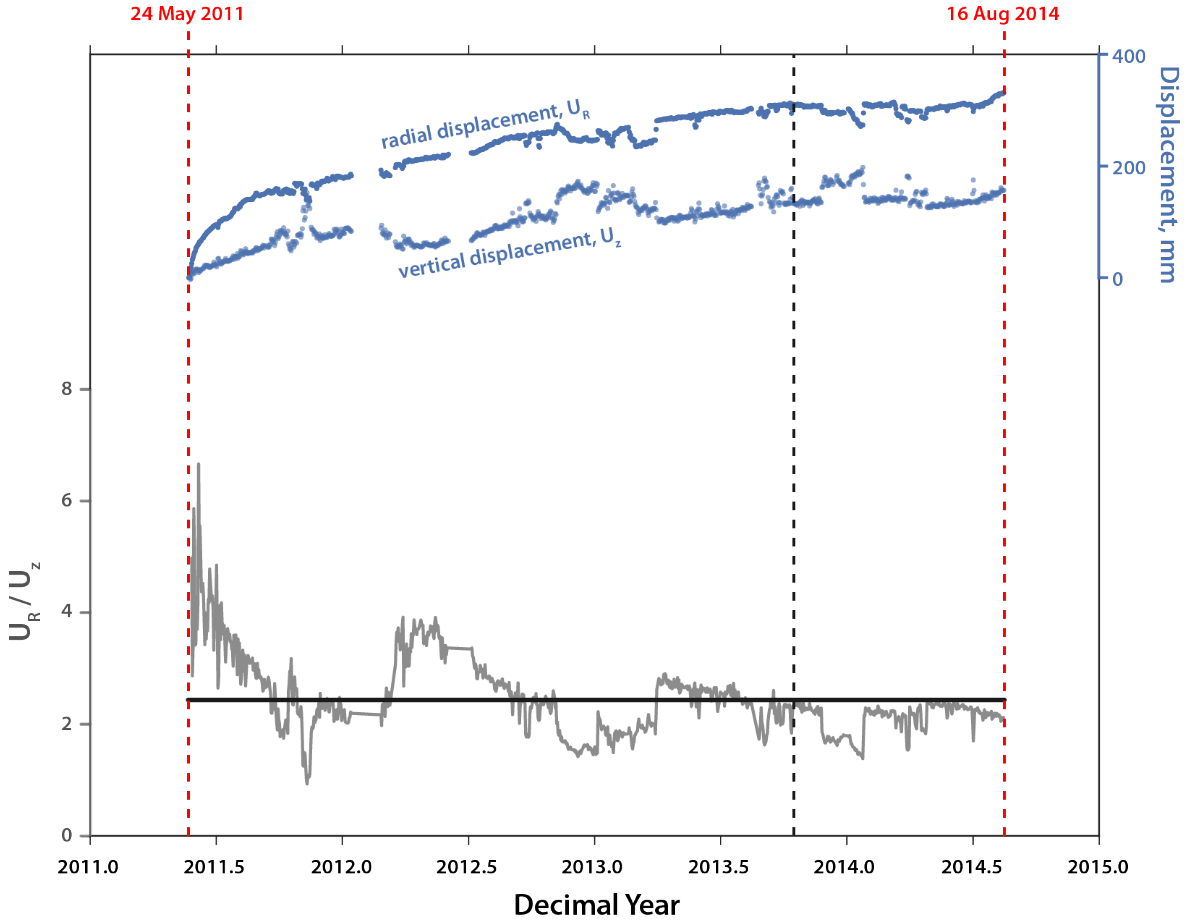

Figure S3: *The ratio of the radial and vertical displacements (gray solid line) at GFUM GPS station from 24 May 2011 to 16 Aug 2014. The horizontal black line is the mean ratio (i.e.* $U_R/U_z = 2.4$*). The blue points are the actual radial and vertical displacements. Tectonic correction is applied on the radial component. The vertical component is neither corrected for GIA nor seasonal effects. The black broken line marks the assumed change of slope discussed in the main text.*

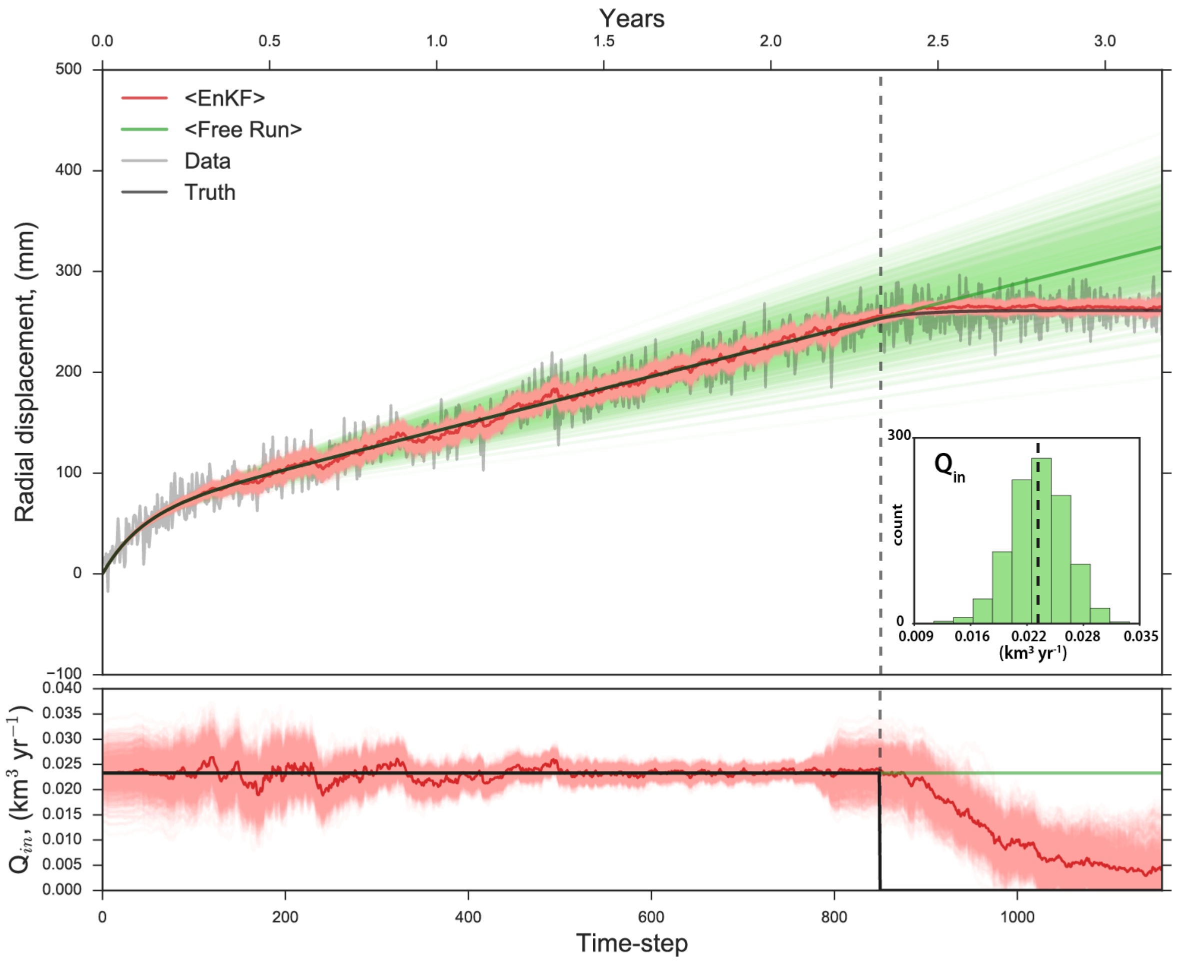

Figure S4: *EnKF synthetic test to track sudden change in Q_{in} value. The true behavior of the system is the black solid line. The radial displacement data used for the EnKF is in gray. The result of free running forward model is in green (the darker green is the mean and the lighter green lines are the ensemble members of the model state), whereas the EnKF result is in red (dark red is the mean value and the lighter red color are the ensemble members of the model state). The vertical black broken lines mark the change of slope (i.e. 875 d). Note that the prior distribution of Q_{in} used for the assimilation is also presented.*

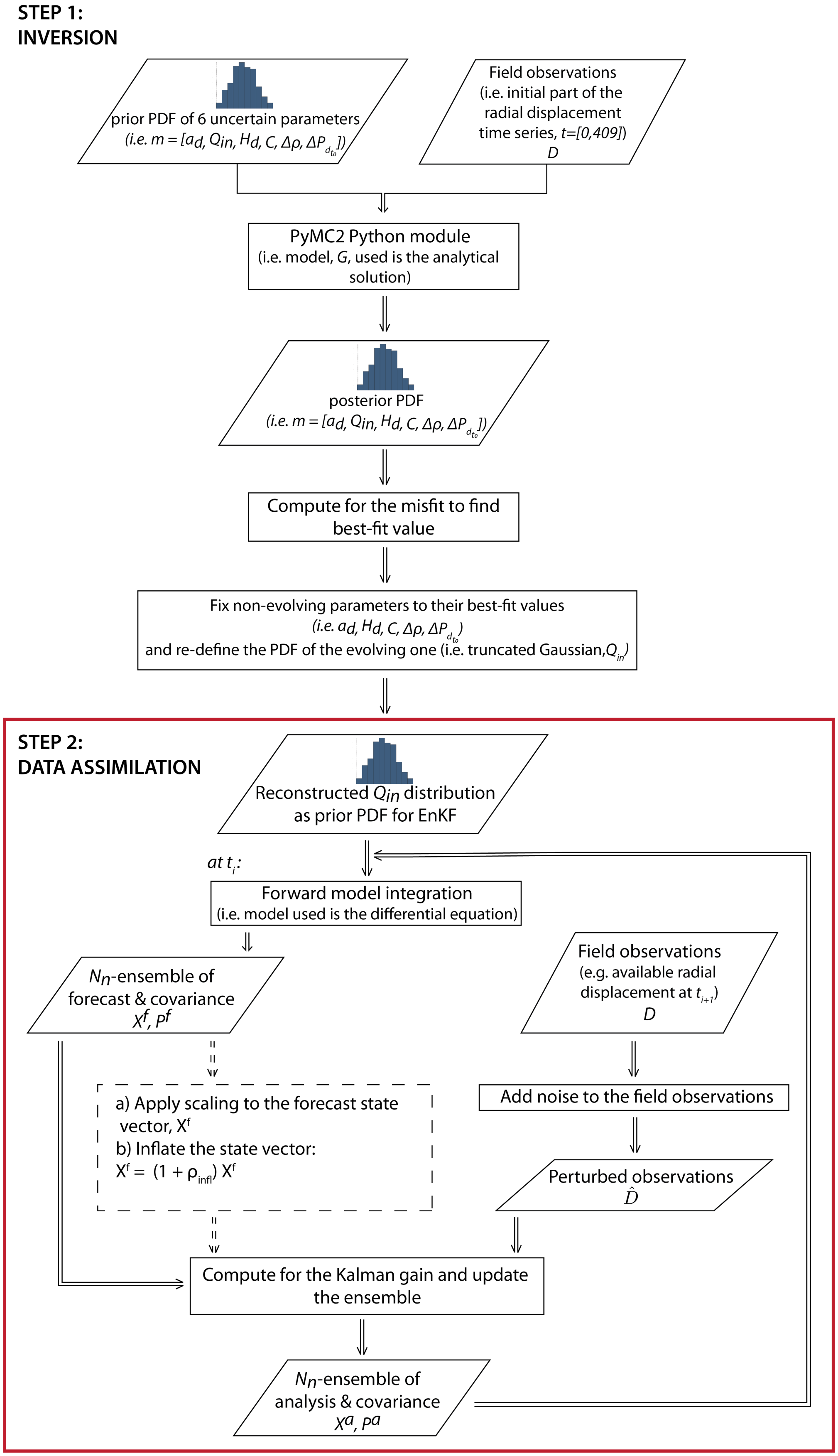

Figure S5: *The two-step strategy (i.e. inversion and data assimilation) implemented in this study. Note that step 2 (i.e. EnKF, enclosed in red box) is modified after ref.[4]. The broken border and lines imply that the step is a tuning step for data assimilation.*

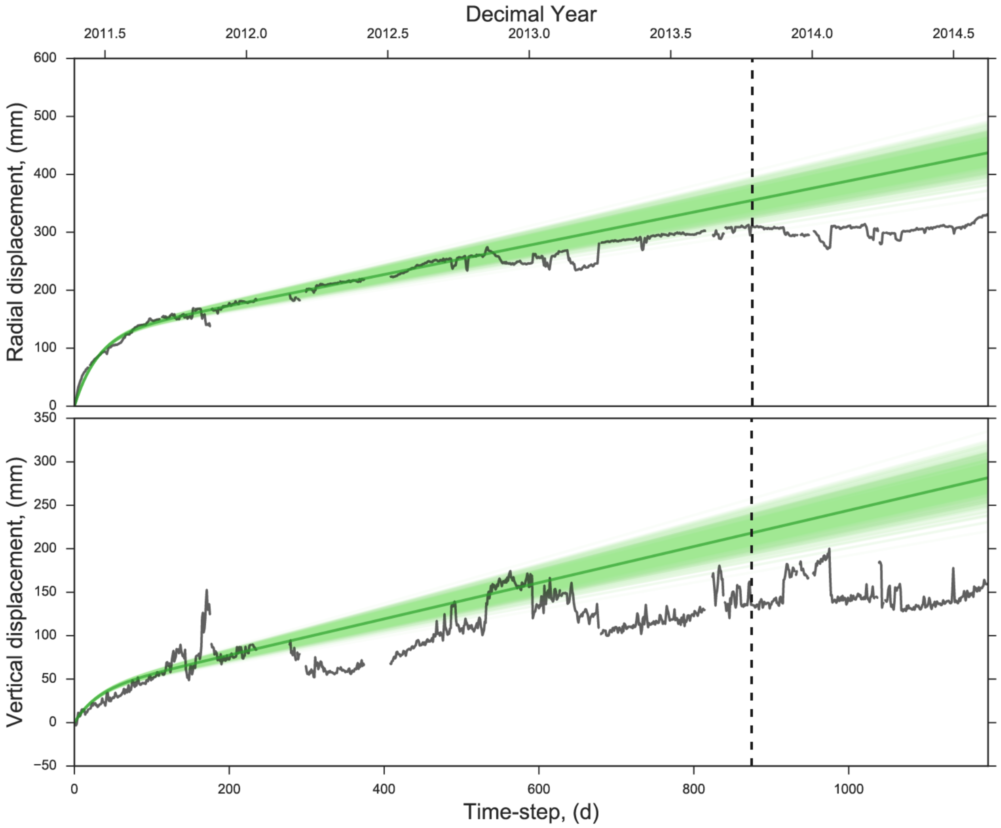

Figure S6: *Data fit (green) using the MCMC-derived values of the uncertain model parameters (see Table 1 in the main text for the best-fit values) as inputs to the forward model. The actual data are represented by gray solid lines. Note that the vertical component is not corrected either for GIA or seasonal effects. The black broken line marks the assumed change of slope preceding the start of the 2014 rifting event* ($t_{step} = 875$ *d).*

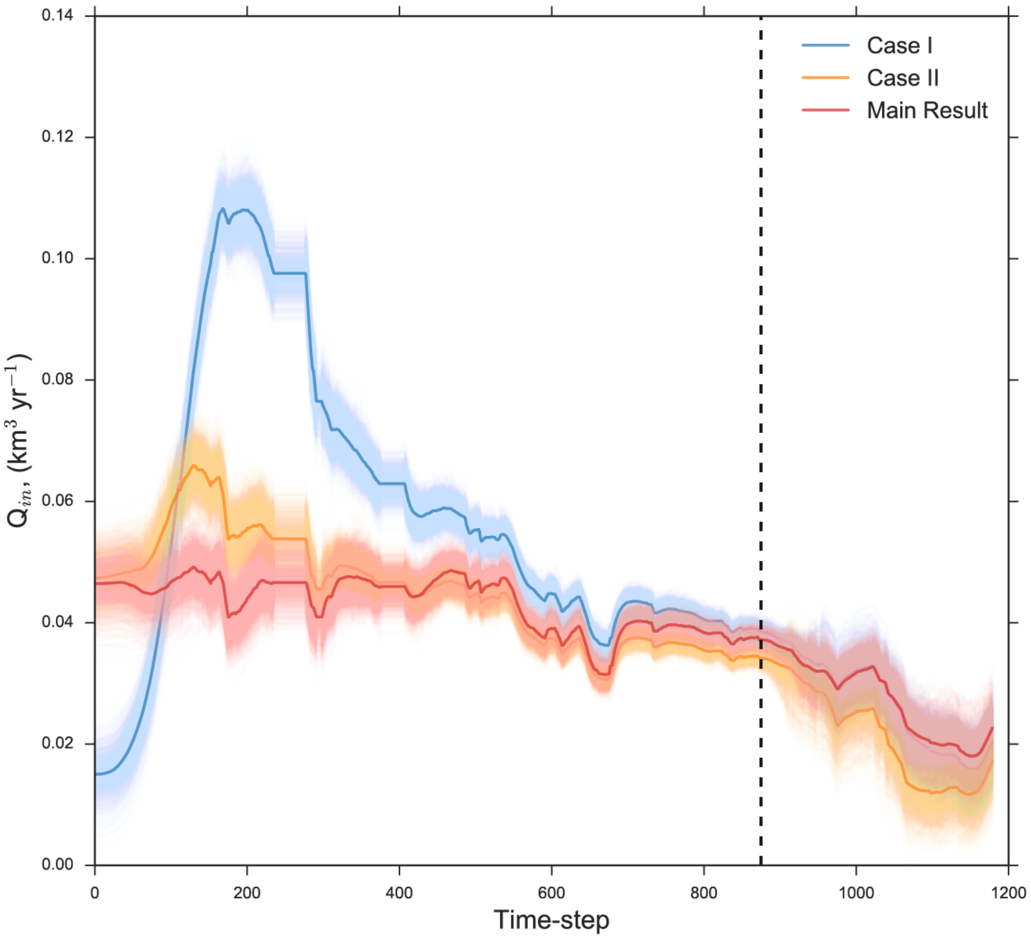

Figure S7: *Testing different sets of uncertain model parameters as prior inputs to EnKF to track the evolution of Q_{in}. Case I: The values of the 6 uncertain parameters are derived using the entire 2004 post-eruptive radial dataset. Case II: Values of a_d, H_d and $\Delta\rho$ from Case I are adopted, whereas the remaining 3 uncertain parameters are determined by inverting the initial part of the 2011 post-eruptive radial dataset. Case III (Main result discussed in the paper): The 6 uncertain model parameters are estimated using the initial part of the 2011 post-eruptive radial dataset. The black broken line marks the assumed change of slope before the start of the 2014 rifting event ($t_{step} = 875$ d).*

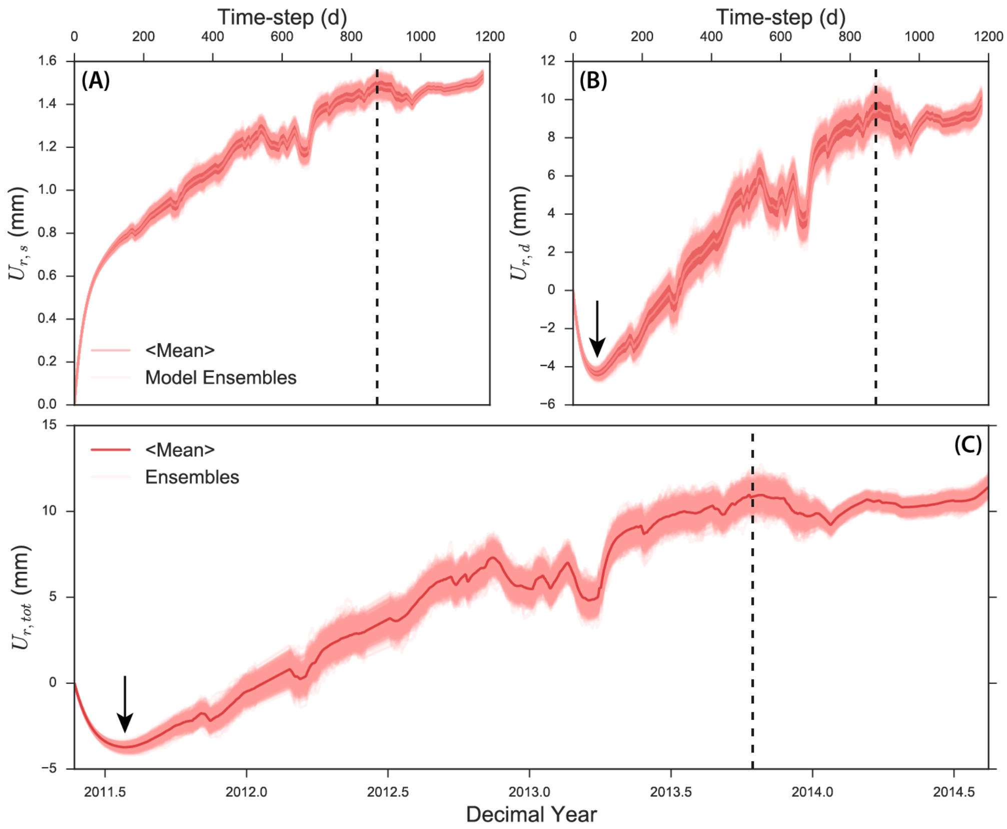

Figure S8: *The expected radial displacement time series measured at a hypothetical GPS station located 15 km away from Grímsvötn's volcanic center. (A) $U_{r,s}$ and (B) $U_{r,d}$ are the radial displacements contributed by the shallow and deep reservoirs, respectively. (C) $U_{r,tot}$ is the combined displacement of the two reservoirs (the data measured at the surface). The black arrow in **Figure S8C** points at the deflected part of the surface displacement curve. This is similarly observed in the radial displacement contribution of the deep reservoir (**Figure S8B**), implying that the measured radial displacement at 15km is mainly dominated by the deep reservoir. The black broken line marks the assumed change of slope prior to the start of the 2014 rifting event ($t_{step} = 875$ d).*

Supplementary Tables

Table S1: *Analyzed displacements at GFUM GPS station at the start of the rifting event (marked as the red broken line in Figure 2 in the main text). Linear Fit corresponds to the expected displacement value (black solid line in Figure 2 in the main text), and "Actual" means the actual displacement value.*

	**Linear Fit**	**Actual**	**Actual - Linear Fit**
u_Y (mm)	-279.03	-259.48	19.55
u_X (mm)	243.99	208.83	-35.16
u_d (mm)	180.13	151.33	-28.80

Table S2: *Description of the parameters and the values at GFUM and DYNC stations used to model the displacements induced by an inflating Bárðarbunga reservoir.*

	**Description**	**GFUM**	**DYNC**	**Ref.**
Y (m)	Distance between the GPS station and Bárðarbunga along NS	-26317.002	20164.004	
X (m)	Distance between the GPS station and Bárðarbunga along EW	7663.885	8727.437	
d (km)	Depth of the Bárðarbunga reservoir	11.0 to 16.0		[2]
R (km)	Radial distance between Bárðarbunga's reservoir and the GPS station	29.54 to 31.74	24.57 to 27.18	
a (km)	Radius of the Bárðarbunga reservoir	3.0 to 4.0		[2]
α	Shape of the reservoir: 1.0 for spherical source, $(4d^2)/(\pi R^2)$ for sill	0.18 to 0.32; 1.0	0.26 to 0.44; 1.0	[2, 3]
ν	Poisson's ratio	0.25		[1]
$\Delta P/E$	Ratio of the overpressure over the Young's modulus	1.95×10^{-5} to 6.67×10^{-4}		[1, 3]
u_Y (mm)	Modeled displacement along NS direction	-65.85 to -0.18	0.34 to 80.34	
u_X (mm)	Modeled displacement along EW direction	0.05 to 19.18	0.15 to 34.77	
u_d (mm)	Modeled displacement along the vertical direction	0.07 to 40.04	0.19 to 63.75	

Table S3: *Analyzed displacements at DYNC GPS station at the start of the rifting event (marked as the red broken line in **Figure S2**). Linear Fit corresponds to the expected displacement value (black solid line in **Figure S2**), and "Actual" means the actual displacement value.*

	**Linear Fit**	**Actual**	**Actual - Linear Fit**
u_Y (mm)	17.63	24.81	7.18
u_X (mm)	172.74	173.45	0.71
u_d (mm)	16.72	17.91	1.19

References

[1] Auriac, A. *et al.* InSAR observations and models of crustal deformation due to a glacial surge in Iceland. *Geophys. J. Int.* **198**, 1329–1341 (2014).

[2] Gudmundsson, M. T. *et al.* Gradual caldera collapse at Bárdarbunga volcano, Iceland, regulated by lateral magma outflow. *Science* **353** (2016).

[3] Coppola, D., Ripepe, M., Laiolo, M. & Cigolini, C. Modelling satellite-derived magma discharge to explain caldera collapse. *Geology* **45**, 523–526 (2017).

[4] Bato, M. G., Pinel, V. & Yan, Y. Assimilation of deformation data for eruption forecasting: Potentiality assessment based on synthetic cases. *Frontiers in Earth Science* **5**, 48 (2017).

[5] Hreinsdóttir, S. *et al.* Volcanic plume height correlated with magma-pressure change at Grimsvotn Volcano, Iceland. *Nature geoscience* **7**, 214–218 (2014).

[6] Reverso, T. *et al.* A two-magma chamber model as a source of deformation at Grímsvötn Volcano, Iceland. *J. Geophys. Res. Solid Earth* **119**, 4666–4683 (2014).

[7] Haddadi, B., Sigmarsson, O. & Larsen, G. Magma storage beneath Grímsvötn volcano, Iceland, constrained by clinopyroxene-melt thermobarometry and volatiles in melt inclusions and groundmass glass. *Journal of Geophysical Research: Solid Earth* n/a–n/a (2017). URL http://dx.doi.org/10.1002/2017JB014067. 2017JB014067.
